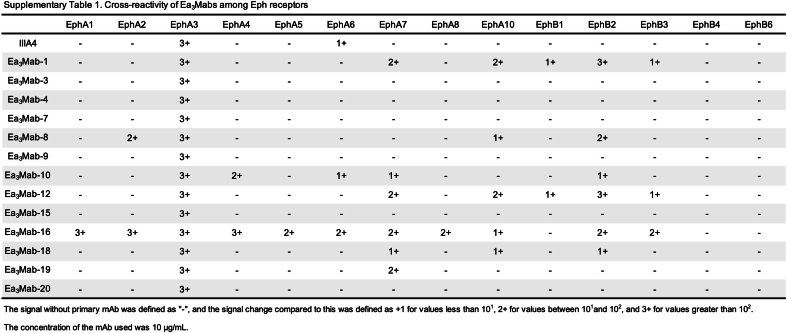# Development of a specific anti-human EphA3 monoclonal antibody, Ea_3_Mab-20, for flow cytometry

**DOI:** 10.1016/j.bbrep.2025.102130

**Published:** 2025-07-04

**Authors:** Hiroyuki Satofuka, Hiroyuki Suzuki, Miu Hirose, Keisuke Shinoda, Takuya Nakamura, Tomohiro Tanaka, Mika K. Kaneko, Yukinari Kato

**Affiliations:** Department of Antibody Drug Development, Tohoku University Graduate School of Medicine, 2-1 Seiryo-machi, Aoba-ku, Sendai, Miyagi, 980-8575, Japan

**Keywords:** EphA3, Monoclonal antibody, Cell-Based Immunization and Screening, Flow cytometry, Immunohistochemistry

## Abstract

Erythropoietin-producing hepatocellular (Eph) receptor A3 (EphA3) is a member of the Eph receptor family, which binds to its respective ligands, ephrins. These interactions are essential for normal development and tissue homeostasis. Dysregulation of EphA3 has been reported to be associated with human hematopoietic malignancies, making it a promising target for therapy and diagnosis. Due to the high similarity of the extracellular domain among Eph receptors (more than 33% amino acid identity), generating highly specific monoclonal antibodies (mAbs) is crucial. We developed anti-human EphA3 mAbs in this study using the Cell-Based Immunization and Screening (CBIS) method. Among them, the clone Ea_3_Mab-20 (IgG_1_, kappa) exhibited high affinity and specificity in flow cytometry. The dissociation constant values of Ea_3_Mab-20 for CHO/EphA3 and Jurkat cells were determined to be 9.0 ± 0.3 × 10^−9^ M and 1.4 ± 0.1 × 10^−9^ M, respectively. Ea_3_Mab-20 showed no cross-reactivity with other Eph receptors in flow cytometry. Furthermore, Ea_3_Mab-20 demonstrated the suitability for detecting formalin-fixed paraffin-embedded cell samples in immunohistochemistry. Therefore, Ea_3_Mab-20 is valuable mAb for basic research and is expected to contribute to the clinical application of mAb for cancer therapy and diagnosis.

## Highlights

• EphA3 represents a promising target for antibody-based cancer therapy and diagnosis.
• A novel anti-EphA3 monoclonal antibody, Ea_3_Mab-20, demonstrated high affinity and specificity in flow cytometry.
• Ea_3_Mab-20 showed no cross-reactivity with other Eph receptors.
• Ea_3_Mab-20 is also available for immunohistochemistry.
• Ea_3_Mab-20 is expected to contribute to clinical application of mAb for cancer therapy and diagnosis.

## 1 Introduction

Erythropoietin-producing hepatocellular (Eph) receptors are a family of receptor tyrosine kinases with a single transmembrane domain, classified into A and B categories based on their extracellular domains [[1], [2], [3], [4], [5], [6]]. The extracellular domains of Eph receptors share a highly similar architecture, consisting of a ligand binding domain, a cysteine-rich region with Sushi and epidermal growth factor-like domains, and two fibronectin type III domains in tandem [[1], [2], [3],^7^], with more than 33% amino acid identity [8]. Eph receptors interact with their membrane-bound ephrin ligands, with each receptor having preferred ephrin ligands [2]. The mammalian Eph system comprises 14 receptor tyrosine kinases (nine EphA and five EphB receptors such as EphA1 to EphA8, EphA10, EphB1 to EphB4, and EphB6) and eight cell surface-anchored ephrin ligands (five glycosylphosphatidylinositol-linked ephrin-As such as ephrin A1 to A5 and three transmembrane ephrin-Bs such as ephrin B1 to B3) [4,9]. These interactions are essential for various normal cellular processes during development and serve as key mediators of adult tissue homeostasis [7,[10], [11], [12]].

The expression of Eph receptors and ephrin ligands can be either upregulated or downregulated in cancer cells compared to normal tissues [5,7,13,14]. Aberrant EphA3 regulation has been reported in human hematopoietic malignancies and solid cancers [5,14,15]. High expression and oncogenic functions of EphA3 have been reported in acute lymphoblastic leukemia (ALL) [16], glioblastoma [17], gastric cancer [18], head and neck cancer [19], and prostate cancer [20]. Conversely, low expression and a tumor-suppressive role of EphA3 have been observed in small-cell lung cancer [21]. Additionally, the correlation between EphA3 mutational status and tumorigenesis in lung cancer has been reported [22]. These findings highlight EphA3 as an important therapeutic target for cancer treatment.

In the development of therapeutic monoclonal antibodies (mAbs) targeting EphA3, the mouse mAb (clone IIIA4) and its humanized defucosylated mAb ifabotuzumab (KB004) were developed to target EphA3-overexpressing cells [7,23]. Ifabotuzumab selectively binds to EphA3-positive cancer cells [24] and can stimulate antibody-dependent cell-mediated cytotoxicity (ADCC) [25]. This agent also prevents the proliferation of cancer cells and endothelial cells in the tumor vasculature by inhibiting EphA3 signaling [26]. Ifabotuzumab was evaluated in a Phase I clinical trial for the treatment of patients with hematological malignancies, demonstrating some encouraging clinical responses [24]. Moreover, treatment with IIIA4, conjugated to maytansine or lutetium-177, prevented tumor formation in glioblastoma-bearing mice [17]. Additionally, EphA3-targeted chimeric antigen receptor (CAR)-T cells demonstrated robust antigen-specific killing of human glioblastoma and diffuse midline glioma cell lines in animal models [27,28]. The EphA3 CAR, consisted of single-chain variable fragments (scFv) derived from the anti-EphA3 mAbs IIIA4 [27] and 3C3-1 [28], has been reported.

Since the development of therapeutic mAbs requires strict specificity to minimize off-target effects caused by cross-reactivity, we aimed to develop anti-EphA3 mAbs with no cross-reactivity to other Eph receptors. We have previously developed several mAbs against various membrane proteins, including Eph receptors, using the Cell-Based Immunization and Screening (CBIS) method [[29], [30], [31], [32], [33]]. The mAbs obtained using this method are prone to recognize conformational epitopes and are suitable for flow cytometry. Furthermore, some of these mAbs also apply to immunohistochemistry, contributing to therapeutic and diagnostic advancements. Therefore, we employed the CBIS method to generate anti-EphA3 mAbs with strict specificity to develop therapeutic and diagnostic agents targeting EphA3.

## 2 Materials and methods

### 2.1 Cell lines

Human glioblastoma LN229, Chinese hamster ovary (CHO)–K1, and mouse myeloma P3X63Ag8U.1 (P3U1) cells were obtained from the American Type Culture Collection (ATCC, Manassas, VA, USA). The human T cell leukemia cell line Jurkat was obtained from the Cell Resource Center for Biomedical Research, Institute of Development, Aging and Cancer at Tohoku University (Miyagi, Japan). The human T cell leukemia cell line MOLT-4 was obtained from the Japanese Collection of Research Bioresources (JCRB) Cell Bank (Osaka, Japan). These cell lines were cultured as described previously [34].

### 2.2 Plasmid construction and establishment of stable transfectants

The gene encoding human *EPHA3* (NM_005233) was obtained from the RIKEN BioResource Research Center (Ibaraki, Japan). The open reading frames, excluding the signal sequences, were subcloned into the pCAG-Ble vector (FUJIFILM Wako Pure Chemical Corporation, Osaka, Japan) with an N-terminal PA16 tag (GLEGGVAMPGAEDDVV) [35] or a MAP16 tag (PGTGDGMVPPGIEDKI) [36], using the In-Fusion HD Cloning Kit (Takara Bio, Inc., Shiga, Japan). The constructed vectors were named pCAG-PA16-EphA3 or pCAG-MAP16-EphA3. The plasmids were transfected into CHO–K1 and LN229 cells, and stable transfectants were established using SH800S cell sorter (Sony Corporation, Tokyo, Japan) to select the highest expression of EphA3-expressing transfectants as described previously [29]. Other Eph receptor-expressed CHO–K1 cells (e.g., CHO/EphA2) were established and the cell surface expression was confirmed as reported previously [29].

The EphA3-knockout Jurkat cells were generated using the clustered regularly interspaced palindromic repeats (CRISPR)/Cas9 system with EphA3-specific guide RNAs (CCTGGCTTACCTTCATTGGA). Knockout cell lines were isolated using the SH800S cell sorter based on the loss of reactivity to an anti-EphA3 mAb IIIA4.

### 2.3 Hybridoma production

All animal experiments were approved by the Animal Care and Use Committee of Tohoku University (Permit number: 2022MdA-001) and carried out per the NIH (National Research Council) Guide for the Care and Use of Laboratory Animals. Two six-week-old female BALB/cAJcl mice (CLEA Japan, Tokyo, Japan) were intraperitoneally immunized with LN229/EphA3 cells (1 × 10^8^ cells) with Alhydrogel adjuvant (2 %, InvivoGen). After three weekly immunizations (1 × 10^8^ cells), a final booster injection (1 × 10^8^ cells) was administered two days before splenocyte harvesting. Hybridomas were generated as described previously [31]. The hybridoma supernatants that were negative for CHO–K1 cells but positive for CHO/EphA3 cells were identified using flow cytometry (SA3800 Cell Analyzer, Sony Corporation). To produce purified mAbs, hybridomas were cultured in serum-free Hybridoma-SFM medium (Thermo Fisher Scientific, Inc.). The culture supernatant was collected, and the mAbs were purified using the Ab-Capture Kit (ProteNova Inc., Kagawa, Japan) according to the manufacturer's instructions.

### 2.4 Flow cytometry

CHO/EphA3 cells were detached using 1 mM ethylenediaminetetraacetic acid (EDTA; Nacalai Tesque, Inc., Kyoto, Japan). Jurkat and MOLT-4 cells were harvested from cultured cell suspensions by centrifugation. The cells were washed with phosphate-buffered saline (PBS) containing 0.1% bovine serum albumin (BSA) as a blocking buffer and incubated with primary mAbs for 30 min at 4 °C. After washing, the cells were stained with Alexa Fluor 488-conjugated anti-mouse IgG (1:2000 dilution; Cell Signaling Technology, Inc., Danvers, MA, USA) for 30 min at 4 °C. Data were acquired using the SA3800 Cell Analyzer and analyzed using FlowJo software (BD Biosciences, Franklin Lakes, NJ, USA).

### 2.5 Determination of dissociation constant values using flow cytometry

CHO/EphA3 and Jurkat cells were treated with serially diluted Ea_3_Mab-20 and IIIA4 (10–0.0006 μg/mL). Subsequently, the cells were stained with Alexa Fluor 488-conjugated anti-mouse IgG (1:200 dilution) for 30 min at 4 °C. The experiment was repeated three times, and the data were collected using the SA3800 Cell Analyzer. The dissociation constant (*K*_D_) values were determined as described previously [29].

### 2.6 Immunohistochemical analysis

Formalin-fixed paraffin-embedded (FFPE) CHO/EphA3 and CHO–K1 cell blocks were prepared using iPGell (Genostaff Co., Ltd., Tokyo, Japan). Staining was performed using the VENTANA BenchMark ULTRA PLUS (Roche Diagnostics) with the recommended protocol and the ultraView Universal DAB Detection Kit.

## 3 Results

### 3.1 Development of anti-human EphA3 mAbs

**Fig. 1:**
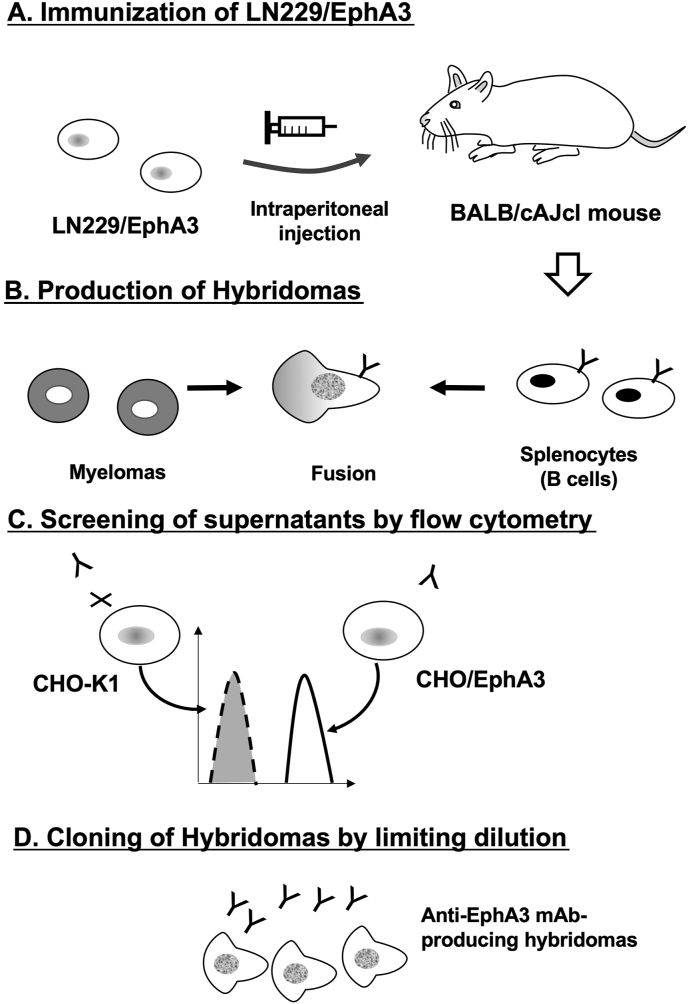
Schematic illustration of anti-EphA3 mAb production using the CBIS method. (A) Two female BALB/cAJcl mice were intraperitoneally injected with LN229/EphA3 cells. (B) Splenocytes were harvested and fused with P3U1 myeloma cells using PEG1500. (C) Hybridoma supernatants were screened by flow cytometry using CHO/EphA3 and parental CHO–K1 cells to identify anti-EphA3-specific mAbs. (D) Hybridoma clones producing antigen-specific mAbs were isolated through the limiting dilution method.

Two BALB/cAJcl mice were immunized with LN229/EphA3 cells (Fig. 1A). After harvesting splenocytes from these mice, cell fusion with P3U1 cells was performed (Fig. 1B). The resulting hybridomas were seeded into 96-well plates. After colony formation, supernatants were collected and analyzed by flow cytometry-based high-throughput screening to identify those that were negative for CHO–K1 cells and positive for CHO/EphA3 cells (Fig. 1C). Subsequently, 20 hybridomas producing anti-EphA3 mAbs were cloned by limiting dilution. Finally, anti-EphA3 mAb clones, including Ea_3_Mab-20 (IgG_1_, kappa), were established (Fig. 1D).

### 3.2 Flow cytometry using Ea_3_Mab-20 and IIIA4

**Fig. 2:**
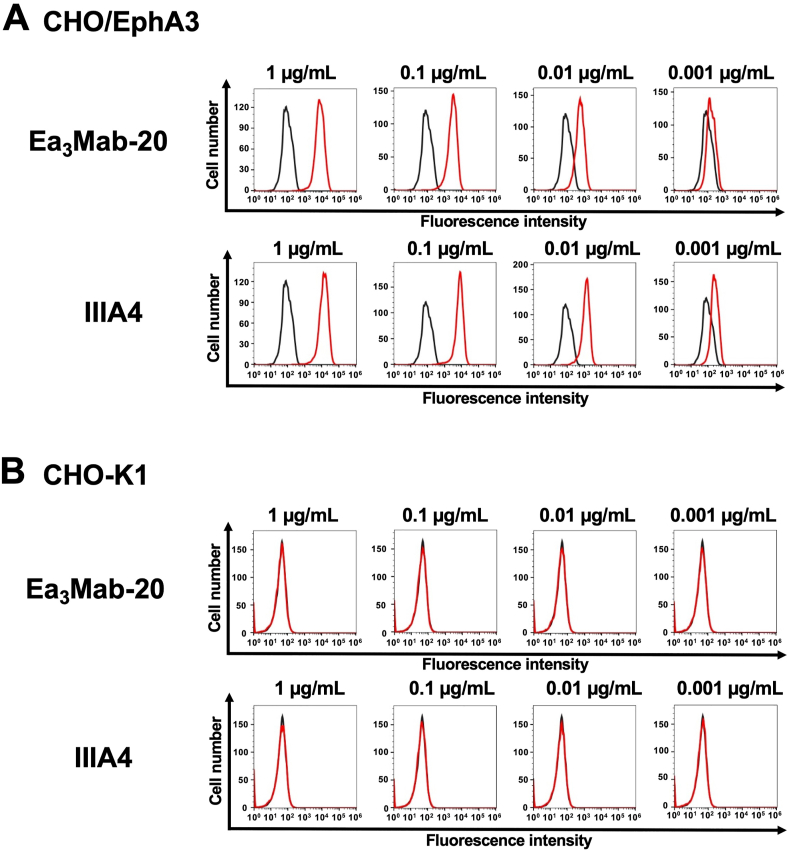
Flow cytometry analysis of anti-EphA3 mAbs against CHO/EphA3 and CHO–K1 cells. CHO/EphA3 (A) and CHO–K1 (B) cells were treated with Ea_3_Mab-20 or the commercially available anti-EphA3 mAb IIIA4 at the indicated concentrations. Cells were stained with (red lines) or without (black lines) anti-EphA3 mAbs, followed by staining with Alexa Fluor 488-conjugated anti-mouse IgG. Fluorescence data were subsequently acquired using the SA3800 Cell Analyzer.

**Fig. 3:**
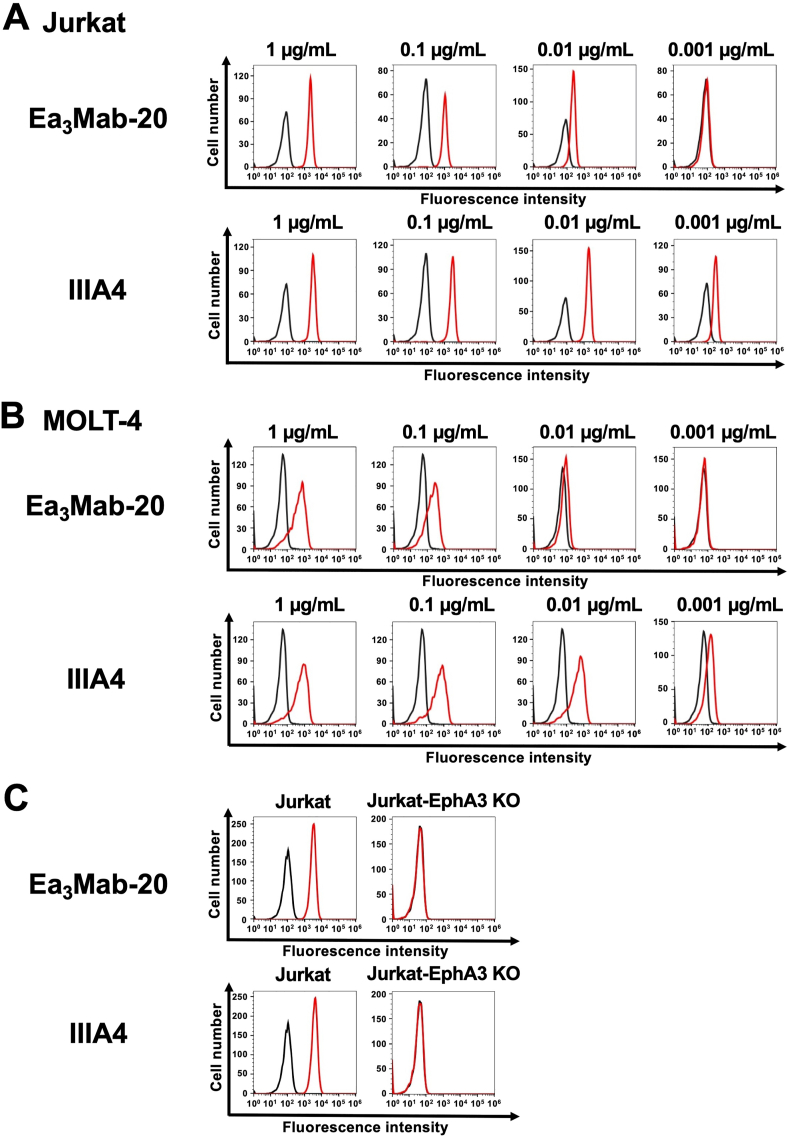
Flow cytometry analysis of anti-EphA3 mAbs against endogenous EphA3 expressing cancer cells. Jurkat (A) and MOLT-4 (B) cells were treated with Ea_3_Mab-20 and IIIA4 at the indicated concentrations (red lines). The black line represents the negative control, which was stained without anti-EphA3 mAbs. (C) Jurkat and EphA3-knockout Jurkat cells were treated with 1 μg/mL of Ea_3_Mab-20, 1 μg/mL of IIIA4, or blocking buffer. The mAb-treated cells were incubated with Alexa Fluor 488-conjugated anti-mouse IgG. Fluorescence data were subsequently collected using the SA3800 Cell Analyzer.

We assessed the reactivity of Ea_3_Mab-20 against CHO/EphA3 and CHO–K1 cells. Ea_3_Mab-20 recognized CHO/EphA3 cells in a dose-dependent manner at concentrations ranging from 1 to 0.001 μg/mL (Fig. 2A). However, Ea_3_Mab-20 did not bind to CHO–K1 cells at any concentrations (Fig. 2B). This result indicates that Ea_3_Mab-20 recognizes EphA3 on the cell surface. A commercially available anti-human EphA3 mAb (IIIA4) exhibited a similar pattern of reactivity with CHO/EphA3 and CHO–K1 cells (Fig. 2). Next, we analyzed the reactivity of Ea_3_Mab-20 against endogenous EphA3-expressing cells, Jurkat (Fig. 3A) and MOLT-4 (Fig. 3B) cells [16], using IIIA4 as a positive control (Fig. 3). Furthermore, to confirm the reactivity of Ea_3_Mab-20 to EphA3, we generated EphA3-knockout Jurkat cells by disrupting the EphA3 gene using CRISPR/Cas9 system (Fig. 3C). The specific reactivity of Ea_3_Mab-20 to EphA3 on Jurkat cells was clearly demonstrated by flow cytometry.

### 3.3 Specificity of Ea_3_Mab-20 using CHO–K1 cells overexpressed various Eph receptors

**Fig. 4:**
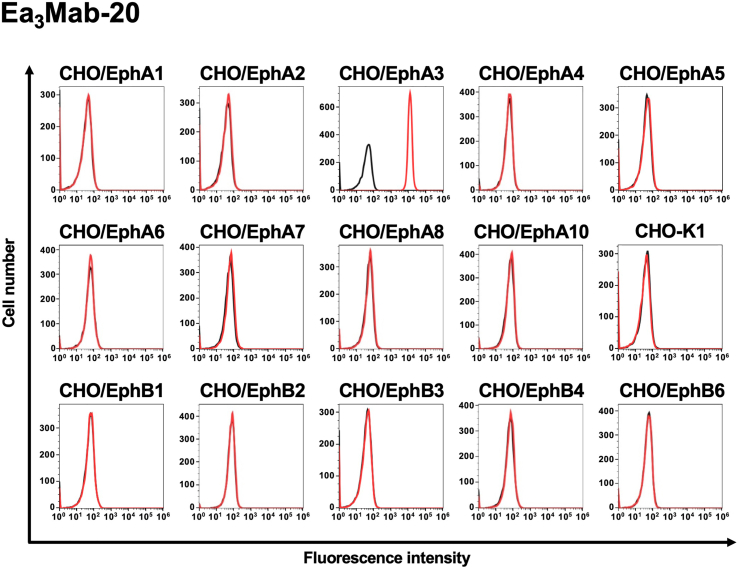
Cross-reactivity of Ea_3_Mab-20 in Eph receptor-expressed CHO–K1 cells. The 14 Eph receptors (EphA1 to A8, A10, B1 to B4, and B6)-expressed CHO–K1 and parental CHO–K1 cells were treated with 10 μg/mL of Ea_3_Mab-20 (red lines). The black line represents the negative control, which was stained without anti-EphA3 mAbs (blocking buffer). The mAb-treated cells were subsequently incubated with Alexa Fluor 488-conjugated anti-mouse IgG. Fluorescence data were collected using the SA3800 Cell Analyzer.

We previously established CHO–K1 cells that overexpressed each human Eph receptor (EphA1 to A8, A10, B1 to B4, and B6) [29]. Using these cell lines, we analyzed the specificity of anti-EphA3 mAbs. Among the 20 clones producing anti-EphA3 mAbs, 13 highly reactive clones were selected for analysis. Staining data with 10 μg/mL of Ea_3_Mab-3, 4, 7, 9, 15, and 20 showed no cross-reactivity among the Eph receptors (Fig. 4 and Supplementary Table 1). However, IIIA4 exhibited slight but significant reactivity with CHO/EphA6 cells (Supplementary Fig. 1), indicating that IIIA4 does not exhibit complete specificity for EphA3. Ea_3_Mab-20 exhibited the highest binding affinity and specificity among these mAbs.

### 3.4 Determination of binding affinity of Ea_3_Mab-20 and IIIA4 using flow cytometry

**Fig. 5:**
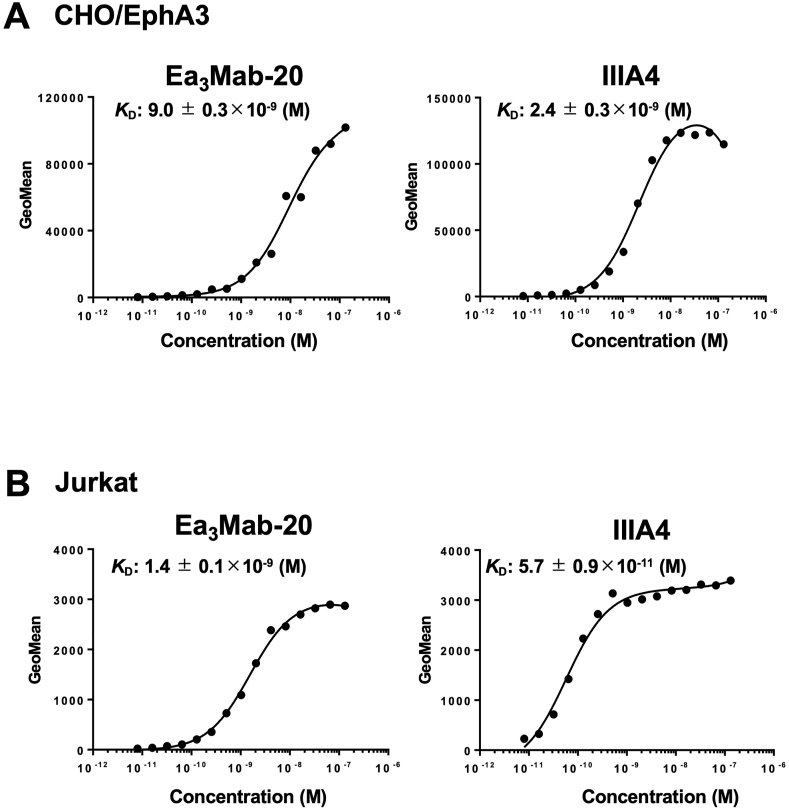
Measurement of the binding affinity of Ea_3_Mab-20 and IIIA4. CHO/EphA3 (A) and Jurkat (B) cells were treated with 100 μL of serial dilutions of Ea_3_Mab-20 and IIIA4 (10–0.0006 μg/mL). Subsequently, fluorescence data were collected using the SA3800 Cell Analyzer. The fluorescence data's geometric mean (GeoMean) values were plotted, and the *K*_D_ values were calculated using GraphPad PRISM 6 software.

The binding affinity of Ea_3_Mab-20 and IIIA4 was determined using CHO/EphA3 and Jurkat cells. The average *K*_D_ values obtained from three independent measurements for Ea_3_Mab-20 were 9.0 ± 0.3 × 10^−9^ M for CHO/EphA3 cells (Fig. 5A and Supplementary Fig. 2) and 1.4 ± 0.1 × 10^−9^ M for Jurkat cells (Fig. 5B and Supplementary Fig. 2). The average *K*_D_ values for IIIA4 were 2.4 ± 0.3 × 10^−9^ M for CHO/EphA3 cells and 5.7 ± 0.9 × 10^−11^ M for Jurkat cells (Fig. 5 and Supplementary Fig. 2).

### 3.5 Immunohistochemistry using anti-EphA3 mAbs

**Fig. 6:**
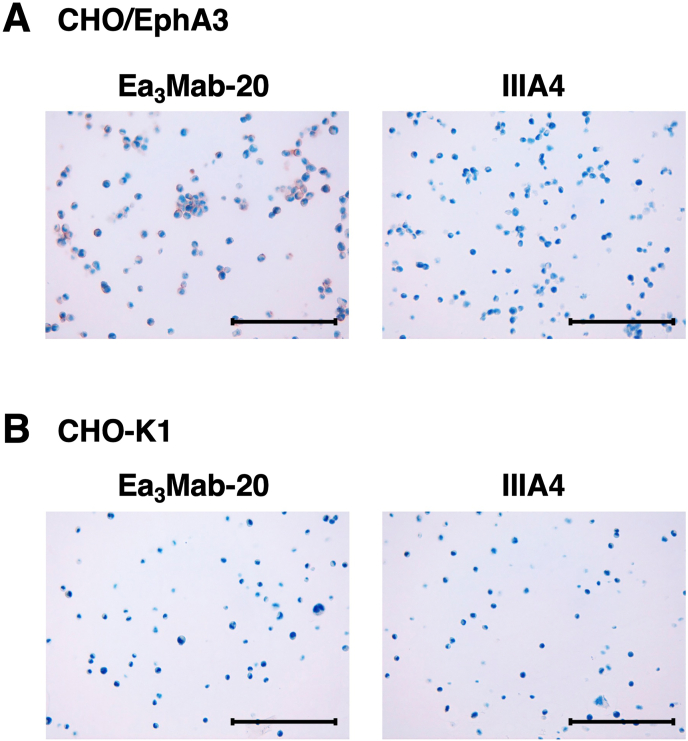
Immunohistochemical staining of paraffin-embedded cell sections of CHO/EphA3 and CHO–K1 cells. Sections of CHO/EphA3 (A) and CHO–K1 (B) cells were stained with 10 μg/mL of Ea_3_Mab-20 and IIIA4 using the VENTANA BenchMark ULTRA PLUS. Scale bar = 100 μm.

Ea_3_Mab-20 was assessed for its application in immunohistochemistry using FFPE CHO–K1 and CHO/EphA3 cell sections. We used the VENTANA BenchMark ULTRA PLUS system for the detection. Apparent membranous staining by Ea_3_Mab-20 was observed in CHO/EphA3 cells (Fig. 6A) but not in CHO–K1 cells (Fig. 6B). In contrast, IIIA4 is unavailable for staining FFPE cell sections (Fig. 6A). In addition, we could not detect endogenous EphA3 in Jurkat cells (Supplementary Fig. 4). These results indicate that Ea_3_Mab-20 is suitable for detecting EphA3-positive cells not only in flow cytometry but also in FFPE samples.

## 4 Discussion

Using the CBIS method, we generated novel anti-EphA3 mAbs, including Ea_3_Mab-20, with confirmed specificity for EphA3. Ea_3_Mab-20 is suitable for flow cytometry (Fig. 2, Fig. 3) and immunohistochemistry (Fig. 6). Furthermore, the specificity of Ea_3_Mab-20 was confirmed using EphA3-knockout Jurkat cells (Fig. 3C). Since Ea_3_Mab-20 does not cross-react with other Eph receptors expressed in CHO–K1 cells (Fig. 4), it is versatile for basic research and is expected to contribute to the development of clinical applications of mAbs for cancer therapy and diagnosis.

The cross-reactivity of IIIA4 with EphA6 may be due to the similarity of the extracellular domain among Eph receptors. The amino acid identity and similarity of the extracellular domain among human Eph receptors range from 33 to 65% and 72–91%, respectively (Supplementary Fig. 3A). The extracellular domain of EphA6 is the most similar in the phylogenetic tree among Eph receptors (Supplementary Fig. 3B), exhibiting a high amino acid identity (64%) and similarity (91%) with that of EphA3. Furthermore, the IIIA4 has been reported to recognize a conformation-specific epitope within the ephrin-binding domain (amino acids 29–202) of EphA3 [37]. The structural similarity of this epitope between EphA3 and EphA6 may explain why an anti-EphA3 mAb IIIA4 cross-reacts with EphA6. Additionally, several clones of the obtained anti-EphA3 mAbs showed cross-reactivity with various Eph receptors, including EphA6 (Supplementary Table 1). Since the development of therapeutic mAbs requires strict specificity to avoid off-target effects caused by cross-reactivity, Ea_3_Mab-20 is a promising candidate for further development as a therapeutic and diagnostic agent.

The binding affinity of IIIA4 is approximately 3.8-fold higher than that of Ea_3_Mab-20 when analyzed using CHO/EphA3 cells (Fig. 5). However, this ratio increases to approximately 24.6-fold when using Jurkat cells endogenously express EphA3 (Fig. 5). This discrepancy may be due to the endogenous expression of EphA6 in Jurkat cells [35], which could have influenced the affinity analysis through the cross-reactivity of IIIA4 with EphA6. The mAbs with high specificity for Eph receptors, such as Ea_3_Mab-20, are crucial for clinical applications, even though the binding affinity of Ea_3_Mab-20 (*K*_D_: 9.0 ± 0.3 × 10^−9^ M) is lower than that of IIIA4 (*K*_D_: 2.4 ± 0.3 × 10^−9^ M) in CHO/EphA3 cells.

Several preclinical studies reported that anti-EphA3 CAR-T cells are effective against glioblastoma [27,28]. Regarding the binding affinity of CARs consisting of scFv, it has been reported that a low-affinity CAR (*K*_D_: 1.4 × 10^−8^ M), which exhibits more than 40-fold lower affinity for CD19 compared to existing scFvs derived from FMC63, enhances CAR-T cell expansion and prolongs persistence in pediatric patients with ALL [38]. Additionally, it has been noted that a faster off-rate, which reflects the rate at which the antibody dissociates from the antigen, is preferred, particularly in CAR-T therapies. The on-rate and off-rate of Ea_3_Mab-20 should be determined. However, its affinity for CHO/EphA3 and Jurkat cells is on the order of 10^−9^ M, which may provide sufficient potential for CAR development.

We have previously developed two methods for epitope mapping: PA insertion for epitope mapping (PAMAP) and RIEDL insertion for epitope mapping (REMAP) [[39], [40], [41], [42], [43]]. These approaches have successfully identified the epitopes of various mAbs, including anti-mouse CD39 mAb (C_39_Mab-1) [39], anti-CD44 mAbs (C_44_Mab-5 and C_44_Mab-46) [40,41], and anti-EGFR mAbs (EMab-51 and EMab-134) [42,43]. Further investigation is necessary to determine the epitope of Ea_3_Mab-20. If Ea_3_Mab-20 targets a linear and non-glycosylated epitope, this finding could facilitate the development of broadly applicable and highly specific mAbs against other Eph family members through peptide-based immunization strategies.

To effectively target EphA3-positive cancer cells using Ea_3_Mab-20 (IgG_1_), generating a class-switched variant with a mouse IgG_2a_ backbone would be beneficial. Additionally, our previous studies demonstrated that defucosylated IgG_2a_ mAbs enhance ADCC activity and exhibit more potent antitumor effects in mouse xenograft models [44,45]. Developing a class-switched and defucosylated version of Ea_3_Mab-20 could improve its therapeutic efficacy against EphA3-positive cancers in preclinical research. Furthermore, it is also essential to evaluate whether Ea_3_Mab-20 demonstrates ADCC activity or drug-induced cytotoxicity when conjugated with cytotoxic agents.

Altogether, Ea_3_Mab-20 reacts with EphA3 without cross-reactivity with Eph family members and is suitable for flow cytometry and immunohistochemistry. EphA3 is a potential therapeutic target, especially in hematopoietic malignancies and brain cancers. Therefore, Ea_3_Mab-20 is a highly sensitive and versatile mAb for basic research and is expected to contribute to clinical applications such as antibody therapy and tumor diagnosis.

## CRediT authorship contribution statement

**Hiroyuki Satofuka:** Writing – original draft, Investigation, Funding acquisition. **Hiroyuki Suzuki:** Writing – review & editing, Investigation, Funding acquisition. **Miu Hirose:** Investigation. **Keisuke Shinoda:** Investigation. **Takuya Nakamura:** Investigation. **Tomohiro Tanaka:** Investigation, Funding acquisition. **Mika K. Kaneko:** Conceptualization. **Yukinari Kato:** Writing – review & editing, Project administration, Funding acquisition, Conceptualization.

## Author disclosure statement

The authors have no conflict of interest in this article.

## Funding information

This research was supported in part by 10.13039/100009619Japan Agency for Medical Research and Development (AMED) under Grant Numbers: JP24am0521010 (to Y·K.), JP24ama121008 (to Y·K.), JP24ama221339 (to Y·K.), JP24bm1123027 (to Y·K.), and JP24ck0106730 (to Y·K.), and by the 10.13039/501100001691Japan Society for the Promotion of Science (JSPS) Grants-in-Aid for Scientific Research (KAKENHI) grant nos. 24K11652 (to H. Satofuka), 22K06995 (to H. Suzuki), 24K18268 (to T.T), and 25K10553 (to Y·K.).

## Declaration of competing interest

The authors declare that they have no known competing financial interests or personal relationships that could have appeared to influence the work reported in this paper.

## Figures and Tables

**Figure:**
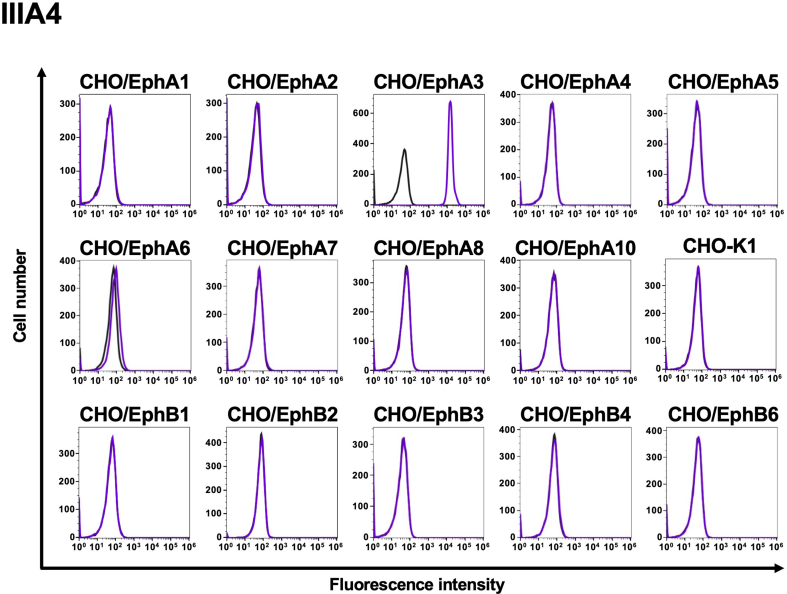
Supplementary figure 1Cross-reactivity of the commercially available anti-EphA3 mAb, IIIA4. The 14 Eph receptors (EphA1 to A8, A10, B1 to B4, and B6)-expressed CHO–K1 and parental CHO–K1 cells were treated with 10 μg/mL of IIIA4 (red lines). The black line represents the negative control, which was stained without anti-EphA3 mAbs (blocking buffer). The mAb-treated cells were subsequently incubated with Alexa Fluor 488-conjugated anti-mouse IgG. Fluorescence data were collected using the SA3800 Cell Analyzer.

**Figure:**
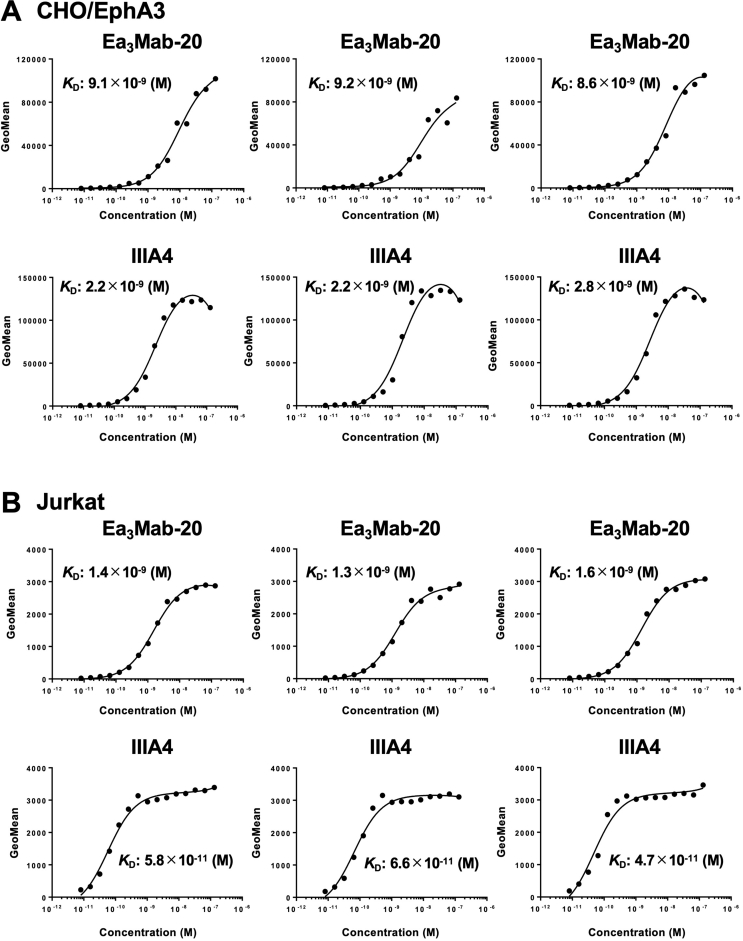
Supplementary figure 2Determination of *K*_D_ values using CHO/ EphA3 (A) and Jurkat (B) cells. Triplicate experiments were conducted, and representative plots are shown in Fig. 5.

**Figure:**
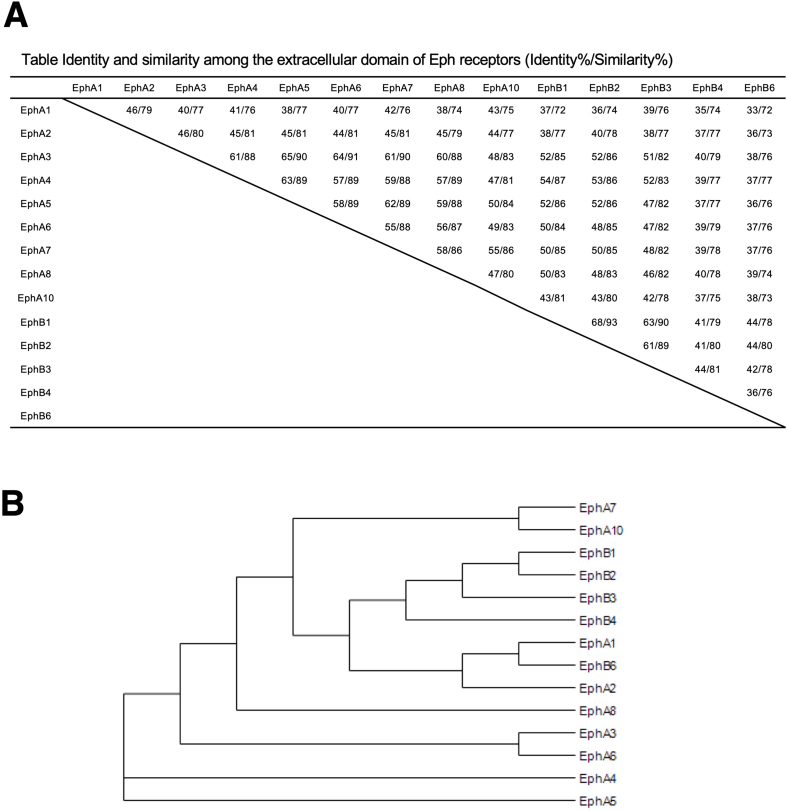
Supplementary figure 3(A) Identity and similarity among the extracellular domains of human Eph receptors. (B) The amino acid sequences of the extracellular domains of Eph receptors (EPHA1, P21709; EPHA2, P29317; EPHA3, P29320; EPHA4, P54764; EPHA5, P54756; EPHA6, Q9UF33; EPHA7, Q15375; EPHA8, P29322; EPHA10, Q5JZY3; EPHB1, P54762; EPHB2, P29323; EPHB3, P54753; EPHB4, P54760; EPHB6, O15197) were extracted from the UniProt database. The phylogenetic tree was generated using the Unweighted Pair Group Method with Arithmetic Mean (UPGMA) clustering based on amino acid sequence similarities among the extracellular domains of Eph receptors by using the Genetyx software (NIHON SERVER Corp., Tokyo, Japan). Branch lengths reflect sequence divergence under the molecular clock assumption.

**Figure:**
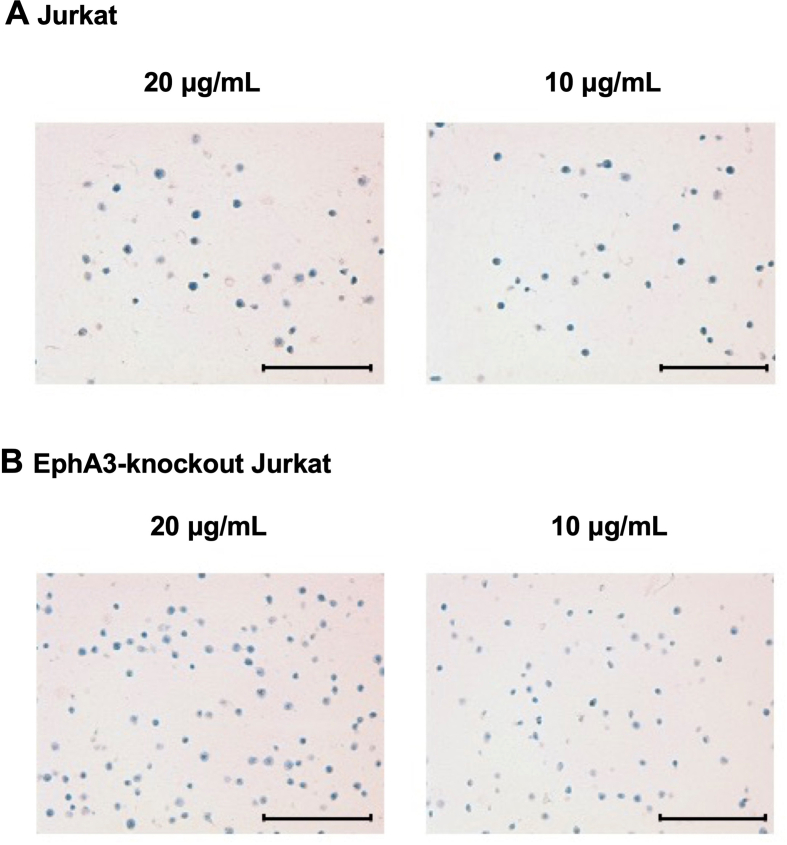
Supplementary figure 4Jurkat (A) and EphA3-knockout Jurkat (B) cells were stained with Ea_3_Mab-20 at indicated concentrations using the VENTANA BenchMark ULTRA PLUS system. Scale bar = 100 μm.

**figs5:**